# Making manual scoring of typed transcripts a thing of the past: a commentary on Herrmann (2025)

**DOI:** 10.1080/2050571X.2025.2514395

**Published:** 2025-06-09

**Authors:** Hans Rutger Bosker

**Affiliations:** Donders Institute for Brain, Cognition and Behaviour, Radboud University, Nijmegen, the Netherlands

**Keywords:** Speech intelligibility, manual assessment, automated scoring, natural language processing, large language models, artificial intelligence, listener transcripts, reproducibility

## Abstract

Coding the accuracy of typed transcripts from experiments testing speech intelligibility is an arduous endeavour. A recent study in this journal [Herrmann, B. 2025. Leveraging natural language processing models to automate speech-intelligibility scoring. *Speech, Language and Hearing, 28*(1)] presents a novel approach for automating the scoring of such listener transcripts, leveraging Natural Language Processing (NLP) models. It involves the calculation of the semantic similarity between transcripts and target sentences using high-dimensional vectors, generated by such NLP models as ADA2, GPT2, BERT, and USE. This approach demonstrates exceptional accuracy, with negligible underestimation of intelligibility scores (by about 2-4%), numerically outperforming simpler computational tools like Autoscore and TSR. The method uniquely relies on semantic representations generated by large language models. At the same time, these models also form the Achilles heel of the technique: the transparency, accessibility, data security, ethical framework, and cost of the selected model directly impact the suitability of the NLP-based scoring method. Hence, working with such models can raise serious risks regarding the reproducibility of scientific findings. This in turn emphasises the need for fair, ethical, and evidence-based open source models. With such models, Herrmann’s new tool represents a valuable addition to the speech scientist’s toolbox.

## Introduction

Doing speech science is hard. Testing how intelligible certain speech recordings are to a given participant often involves the meticulous scoring of written transcripts (for instance, collected in ‘type out what you hear’ tasks). This scoring is difficult, time-consuming, and often simply outright boring. Research tools that can alleviate part of this experience are therefore highly desirable.

Two automated scoring applications were already available to speech researchers. One is Autoscore (Borrie et al., 2019), a tool that, at the most basic level, counts words in transcripts as correct if they exactly match the words in the target sentence. It performs well, is very fast, is available as an R package, and also has an online user interface for less code-savvy users (http://autoscore.usu.edu/). However, Autoscore is not readily applicable to languages other than English and requires manually adapted spelling lists to handle common misspellings. Then there is the Token Sort Ratio (TSR; Bosker, 2021), a fuzzy string matching technique that quantifies the match between an orthographic transcript and the target sentence based on the number of shared characters. This technique is also fast, demonstrates high correlations with human scores, and is available as a Python script as well as an open online user interface (https://tokensortratio.netlify.app/). Moreover, by quantifying orthographic similarity, it is more lenient towards slight misspellings and can be applied to any language. Nevertheless, in the lower end of the intelligibility range, the TSR tends to overestimate the accuracy of listener transcripts.

Now Herrmann (2025) presents a promising innovation by making use of Natural Language Processing models (NLP; also known as Large Language Models, LLM) to automate the assessment of listener transcript accuracy. Herrmann’s approach quantifies the match between a typed transcript and a target sentence by calculating the Spearman correlation between high-dimensional vectors of the transcript and target sentence, as generated by NLP models. This correlation captures semantic similarity rather than relying on shared orthography or exact word matches, providing a continuous measure of intelligibility that reflects how closely the response aligns with the meaning of the target sentence.

Specifically, Herrmann (2025) used previously acquired speech-intelligibility data (Herrmann, 2023; Experiments 1–2) collected from 144 younger and older participants. These participants listened to short, phonetically balanced English sentences (Harvard sentence lists 1–15) in various levels and types of noise and were asked to type out what they heard. Their transcripts and the target sentences were mapped onto high-dimensional vectors of real numbers (i.e., embeddings) using different large language models, including ADA2, GPT2, BERT, and USE. The NLP-based scores, namely the Spearman correlations between these two types of vectors, correlated at around .95 with manual scores of transcript accuracy. Moreover, across all models, there was only negligible underestimation of intelligibility scores (by about 2–4%). This is a particularly impressive achievement, signifying very high accuracy, and numerically outperforming simpler computational tools. Thus, the NLP-based scores were able to accurately capture well-established general age-related reduction in intelligibility as well as age-related reduction in the perceptual benefit from a modulated vs. unmodulated masker (Herrmann, 2025).

A unique aspect of NLP-based scoring is its reliance on semantic representations, which reflect the end-goal of human speech comprehension, namely mutual understanding. Interestingly, many tests of speech intelligibility focus rather on word identification as index of speech comprehension. Hence, the transcript ‘Rachel could play the piano’ for the sentence ‘Rachel could not play the piano’ may receive a higher score from human scorers of speech intelligibility than from the NLP-based approach. This raises important questions about our tests, our tools, and their alignment: do we want to assess speech intelligibility in terms of word identification, or speech understanding, which may fail, like in the above example, even if most words were correctly identified (for a relevant review, see Baese-Berk et al., 2023)? That said, the remarkably high accuracy of the NLP scores arguably indicates that, for this type of research design, misalignment of speech intelligibility and speech understanding is rare.

Also, Herrmann’s tool uniquely relies on NLP. The consistency with which different LLMs (here: ADA2, GPT2, BERT, USE) correctly captured listener transcript accuracy is remarkable, promising cross-model generalizability of the applicability of NLP-based scoring. LLMs are swiftly growing and developing and hence we may even see further improvement of the NLP-based scoring technique. This also adds to its cross-linguistic applicability: more and more LLMs are becoming available in a diverse range of languages and hence the technique could be applied to data in different languages, providing a significant advantage for cross-linguistic research.

Critically, the reliance on LLMs is a unique strength, yet at the same time also the Achilles heel of the NLP-based scoring approach. Variations and/or restrictions in the accessibility, security, accountability, ethical profile, and cross-linguistic variability of LLMs directly impact the accessibility, security, accountability, ethics, and applicability of the new scoring technique. Interestingly, many concerns have been identified regarding these aspects of LLMs (Liesenfeld et al., 2023).

For instance, OpenAI’s ADA2 was identified in Herrmann (2025) as the best-performing model in terms of accuracy and also demonstrated good running time efficiency. These arguments could motivate researchers, who plan to implement the NLP-based scoring approach in their own study designs, to select ADA2 for their data scoring. However, ADA2 is only *accessible* through an account with OpenAI and an application programming interface (API) key. Hence, *data security* is a serious concern, especially when working with vulnerable (clinical) populations. Also, using ADA2 comes with a *cost*, although these are – at present – only minimal. Furthermore, even though ADA2 can handle input from languages other than English, it was primarily trained on English data. Therefore, its performance may be reduced when working with other (e.g., underrepresented) languages, *restricting its cross-linguistic applicability*. Finally, there are also serious concerns around *reproducibility* – one of the core values of science. For instance, OpenAI has already presented a replacement LLM for ADA2, text-embedding-3-small, with ADA2 deprecation and retirement scheduled around October, 2025; Microsoft Azure, n.d.). This entails that ADA2, in the future, will no longer be available for use, returning error responses. This would severely hurt reproducibility of scientific work, which is already being undermined by deprecation of formerly widely used LLMs (e.g., OpenAI’s davinci; Liesenfeld & Dingemanse, 2024).

All these considerations emphasise the vulnerability of the NLP-based scoring technique to developments and changes in the terms and conditions, quality, accessibility, data security, ethical profile, and carbon footprint of different LLMs; a vulnerability that does not apply to the two alternative tools (i.e., Autoscore, TSR). Consequently, researchers should be cautious when selecting this NLP-based technique and specifically when selecting a model to calculate the NLP-based scores. In this light, the fact that the NLP-based approach seems robust across different LLMs is promising. There is considerable variation in how open, accessible, secure, and reproducible different LLMs are (e.g., USE, GPT2, and BERT are more open than ADA2; Liesenfeld et al., 2023). One should use maximally open, non-proprietary models to ensure transparency and independence (Widder et al., 2023); work locally, never sharing any personal, privacy-sensitive data outside one’s research institute; think ahead about the reproducibility of the obtained scores and be ready to be held accountable by reviewers and scientific peers; follow the institutional (for example, see Dingemanse, 2024) and national guidelines (e.g., the EU’s GDPR) regarding responsible AI use; and be prepared to justify any choices in response to an ethics review board or a GDPR access request.

All in all, we see that it’s indeed hard work doing speech science. Fortunately, in today’s day and age, there’s a growing body of resources to make our research easier. Through online testing and large-scale participant banks, experimental data sets can be collected much more efficiently, growing in size and boosting their representativeness. In turn, the larger size of our data sets calls for automatic tools that lighten the burden of manual scoring. Herrmann’s (2025) NLP-based scoring approach adds another efficient new tool to the speech scientists’ toolbox. That said, working with NLP models entails significant risks around data security, ethics, and reproducibility. Therefore, responsible application of the NLP-based scoring technique involves employing models that are open, transparent, and accountable, thus upholding research quality and integrity.
